# Personality Trait Profiles in People With Mild Intellectual Disability: A Comparative Study

**DOI:** 10.1111/jir.70032

**Published:** 2025-08-07

**Authors:** Renate A. van der Heijden, Paul T. van der Heijden, Hubert P. L. M. Korzilius, Han Berghuis, Robert Didden

**Affiliations:** ^1^ Centre for Psychiatry and Mild Intellectual Disability GGZ Oost Brabant Boekel The Netherlands; ^2^ Behavioural Science Institute Radboud University Nijmegen The Netherlands; ^3^ Reinier van Arkel Mental Health Institute 's‐Hertogenbosch The Netherlands; ^4^ Institute for Management Research Radboud University Nijmegen The Netherlands; ^5^ NPI, Centre for Personality Disorders Amersfoort The Netherlands; ^6^ Trajectum Zwolle The Netherlands

**Keywords:** behavioural measurement methods, borderline intellectual functioning, health inequities, intellectual disability, mental health, personality disorders

## Abstract

**Background:**

Personality assessment in people with mild intellectual disability (MID) is difficult due to their communication difficulties and lack of reliable instruments. In addition, characteristics of maladaptive personality traits may be attributed to the intellectual disability. As a result, little is known about (maladaptive) personality traits in people with MID. The aim of this study was to explore maladaptive personality traits of people with MID and compare them to those of two comparison groups.

**Methods:**

Maladaptive personality traits of people with MID referred to specialised mental health care (*n* = 75) were compared with those of people with borderline intellectual functioning referred to specialised mental health care (BIF, *n* = 69) and those of people with average educational levels from general mental health care (AVE, *n* = 73) using the Dimensional Assessment of Personality Pathology–Short Form.

**Results:**

People with MID scored higher on Affective Lability, Anxiousness, Identity Problems, Insecure Attachment and Suspiciousness and lower on Narcissism and Social Avoidance compared to the people with AVE. No differences in personality trait scores were found between people with MID and people with BIF, except for a lower score on Social Avoidance in those with MID. Almost all differences demonstrated small effect sizes.

**Discussion:**

Maladaptive personality traits of people with MID and comorbid psychopathology are of similar severity compared to those of people with BIF and comorbid psychopathology or people in mental health care with average educational levels. This study emphasises that clinicians look beyond the intellectual and adaptive disabilities when assessing for mental health problems in people with MID, while meeting their needs when it comes to the treatment of these problems.

## 1 Introduction

People with mild intellectual disability (MID) have an increased risk of developing psychopathology. Research shows that approximately one third of people with MID have a comorbid mental disorder (Mazza et al. 2020). Consequently, people with MID are highly present in mental health care services (Nieuwenhuis et al. 2017; Seelen‐de Lang et al. 2019). The increased risk may be explained by the higher number of psychological and social risk factors compared to people with average intellectual functioning, such as stressful life events (Bond et al. 2019), (very) low income, poor family functioning and social exclusion (Cooper 2020). Additionally, people with MID have fewer abilities to cope with these events due to intellectual and adaptive impairments (Cooper 2020) and might show less help‐seeking behaviour (Koenen et al. 2009), resulting in more severe psychopathology, including maladaptive personality traits.

Maladaptive personality traits refer to (a dimensional approach of) personality characteristics that have a negative or maladaptive effect on an individual's functioning (Altschuler and Krueger 2021). Several dimensional models for maladaptive personality traits exist, including the model of the ICD‐11 (World Health Organisation 2022) and the Alternative Model for Personality Disorders in the DSM‐5‐TR (AMPD; APA 2022). These dimensional models have a common hierarchical structure that contains five broad maladaptive personality trait domains: Negative Affectivity/Emotional Dysregulation, Antagonism/Dissocial Behaviour, Detachment/Inhibition, Disinhibition versus Compulsivity/Anankastia and sometimes Psychoticism (e.g., Gutiérrez et al. 2019; Wright and Simms 2014). Within these broad personality trait domains, different personality trait facets are distinguished, such as Anxiousness and Affective Lability in the trait domain of Negative Affectivity/Emotional Dysregulation and Withdrawal and Intimacy Problems in the Detachment/Inhibition domain (e.g., Berghuis et al. 2017). These personality trait facets contain more nuanced information and provide more distinguished personality profiles than the broader (maladaptive) trait domains (Ratchford et al. 2022).

Assessing maladaptive personality traits in people with MID is challenging. First, there is a paucity in research on the validity and reliability of (self‐report) personality instruments in people with MID. The common assumption is that people with MID cannot reliably and validly be assessed with self‐report due to intellectual difficulties in understanding and responding to questions regarding their own thoughts, feelings, motives and behaviour. In addition, there is a risk that characteristics of maladaptive personality traits are assigned to the intellectual and adaptive impairments pertaining to MID, which is known as diagnostic overshadowing (Dell'Armo and Tassé 2024). For example, difficulties in controlling emotions such as anger are often (mis)interpreted as emotional immaturity befitting to the MID instead of as a characteristic of a personality disorder (Gentile et al. 2022). In addition, the opposite might also occur in which characteristics of an (unidentified) intellectual disability are interpreted as maladaptive personality traits.

To date, most research on maladaptive personality traits in people with MID has been aimed at the prevalence and validity of categorical personality disorder diagnoses (see Cooray et al. 2022). Recent insights concerning personality disorders lead to a shift from a categorical to a dimensional approach. This can give a new impulse to scientific research and psychological assessment with a focus on maladaptive personality traits, also in people with MID. Advantages of dimensional assessment of personality disorders by means of maladaptive personality traits are the increased reliability, validity, prediction of prognosis and decreased stigmatisation (Hopwood et al. 2018). The dimensional approach might provide more insight into the vulnerabilities and strengths in the personalities of people with MID, without using stigmatising categorical diagnoses. Health care professionals have also recognised that employing a person‐centred approach in the assessment of maladaptive personality traits is less stigmatising, thereby reducing barriers to accessing (mainstream) care, which are often already elevated due to the existing MID classification (Zarotti et al. 2022).

To date, two studies have been published in which dimensional personality traits in people with MID were assessed. In these studies, people with MID and people with borderline intellectual functioning (BIF) were taken together and were compared with a mental health care sample of people with an average educational level. The combined MID‐BIF sample demonstrated higher scores on emotional dysregulation and obsessionality (Lie Sam Foek‐Rambelje et al. 2024) and lower scores on the capacity for mentalisation and reflective functioning (Haacke et al. 2025) than the sample of individuals with average education. However, these studies used the Shedler‐Westen Assessment Procedure, a clinician‐rated instrument to measure personality traits, categorical personality disorders and mental functions such as the capacity for mentalisation (Westen and Shedler 1999). Even though clinician‐rated or other informant‐rated instruments can be very informative, self‐report remains a critical source of information in the assessment of personality trait facets (e.g., Emery and Simms 2023; Oltmanns and Oltmanns 2021; Widiger et al. 2024).

The aim of the current study was to compare self‐reported personality trait facets of a sample of people with MID group referred to specialised mental health care to those of people with BIF group from specialised mental health care, and a comparable mental health care sample consisting of individuals with average educational levels (AVE group). Comparing these three groups will offer valuable insight into how maladaptive trait profiles manifest across different levels of functioning, helping to improve diagnostic perspectives and strategies regarding the assessment of maladaptive personality traits in individuals with MID.

## 2 Method

### 2.1 Setting

The internal research review board of two participating mental health services and the ethics committee of Radboud University, Nijmegen, the Netherlands (21U.005021‐ECSW‐2021‐029) approved the study protocol, documents and procedures. Data of the MID and BIF groups were collected from September 2021 until November 2024. These participants were referred to one of two mental health care services, specialised in the assessment and treatment of psychopathology in people with MID or BIF in the southern region of the Netherlands. Allocation to the MID or BIF group was based on DSM‐5(TR) diagnosis made by clinicians (i.e., licensed psychologist or psychiatrist). Diagnosis was based on diagnostic assessment of adaptive and intellectual functioning using standardised testing as well as observations and interviews.

### 2.2 Procedure

Data were collected in the context of diagnostic assessment and treatment planning. Patients suffering from acute psychosis or otherwise distorted in thinking (e.g., recent substance abuse and delirium) do not follow the standard care procedure following referral and were not included in the current investigation for this reason. Potential participants were informed about the research 2 weeks before the assessment. Written and informed consent was obtained from all participants in the MID and BIF groups at the start of assessment. A clinician or research assistant was available to assist participants to complete the Dimensional Assessment of Personality Pathology (DAPP) if the participants had any questions. Based on their preference, instructions and items were read together with the participant or were given verbatim. Only if necessary, alternatively scripted items were used, in which difficult words were substituted by simplified synonyms, which participants requested in an average 9% of the items. In addition, a show card was used containing the response scale in words (very unlike me to very like me), numbers (one to five) and colours (light blue to dark blue).

### 2.3 Participants

The AVE group consisted of people with average educational levels from an inpatient and outpatient mental health care service. Data were obtained from a dataset used in a study by Berghuis et al. (2017), which originally included 350 participants referred to several mental health services in the Netherlands (for information on the data collection, see Berghuis et al. 2017). For the purpose of the current study, a group with participants with average educational levels was created by selecting only those participants who completed further education (i.e., secondary vocational education; *n* = 249). From this sample, 28 participants were excluded due to missing scores, retaining 221 participants, from which a random sample of 73 participants was drawn. This was done to balance the number of participants in the MID and BIF groups to reduce the risk of violating the assumptions of statistical analyses for group comparisons (Midway et al. 2020).

The sample of people with MID consisted of 75 participants, of whom 45 (60%) self‐identified as female and who had an average age of 37.4 (SD = 12.3). The sample of people with BIF consisted of 69 participants, of whom 45 (65%) were female, with an average age of 37.7 (SD = 13.5). Finally, the sample of people with AVE consisted of 73 participants, of whom 51 (70%) self‐identified as female, with an average age of 34.8 (SD = 10.8).

### 2.4 Instrument

In the current study, the DAPP (Livesley et al. 1992) was used as it is a widely used self‐report instrument compatible with the contemporary dimensional models of personality traits (i.e., DSM‐5‐TR/AMPD and ICD‐11) (Aluja et al. 2024; Berghuis et al. 2017; Gutiérrez et al. 2019). The items of the Dutch language version of the DAPP are compatible with the reading level at the end of Dutch primary school (approximately 12 years of age), which should be feasible for people with MID. In the current study, the term (maladaptive) personality trait domains was used for the DAPP higher order factors, while the term (maladaptive) personality trait facets was used for the lower order factors.

In participants with MID and those with BIF, the DAPP—Short Form (DAPP‐SF; van Kampen et al. 2008) was used, while the AVE group had completed the DAPP—Basic Questionnaire (DAPP‐BQ). Data on the DAPP‐BQ in the AVE group were converted into DAPP‐SF scores in the same way as in the DAPP‐SF development study by van Kampen and colleagues. In this study, the DAPP‐BQ items most defining for a specific factor and of good reliability, without being redundant, were extracted to form the shortened version. Therefore, as the DAPP‐SF is an item subset of the DAPP‐BQ. In the AVE group, the reduced subset was used to calculate new DAPP‐SF facet scores. The DAPP‐SF contains 136 items that are scored on a 5‐point Likert scale ranging from *Very unlike me* (1) to *Very like me* (5). The instrument is organised into four higher order maladaptive personality trait domains (i.e., Emotional Dysregulation, Dissocial Behaviour, Inhibition and Compulsivity). Within these domains, 18 maladaptive personality trait facets are assessed, which are described in Table 1.

**TABLE 1:** DAPP personality trait domains and facets.

Domains	
Trait facets	Facet description
Emotional dysregulation	
Affective lability	Unstable emotions and frequent and rapid mood swings
Anxiousness	Constant anxiety, nervous tension and worry
Cognitive distortion	Disorganised thinking at times of stress and high levels of discomfort
Identity problems	Unstable self‐image or identity structure
Insecure attachment	Fear of rejection, abandonment or loss of relationships with attachment figures and significant others
Narcissism	Feelings of greatness and need for attention
Oppositionality	Passive resistance to expectations, passive interpersonal style and antagonising others
Social avoidance	Avoidance of social relationships and interaction
Submissiveness	Obedient and unassertive attitude and need to look for others for support, advice and reassurance
Suspiciousness	Distrust of the intention of others
Dissocial behaviour	
Callousness	Indifference to the feelings and well‐being of others
Conduct problems	Behavioural problems and antisocial behaviour
Rejection	Hostile, unfriendly and critical behaviour
Stimulus seeking	Need for excitement with reckless and impulsive tendencies
Inhibition	
Restricted expression	Avoidance of showing emotions or revealing (even insignificant) personal information
Intimacy problems	Avoidance of intimacy and close attachment
Compulsivity	
Compulsivity	Preoccupation with order and accuracy and need for structure and organisation
Other	
Self‐harm	Deliberate self‐harm and chronic suicidal ideation

*Note:* Descriptions as found in the Dutch language DAPP manual (van Kampen and de Beurs 2020).

The reliability of the Dutch language version of the DAPP‐SF was found to be good in a community sample (Cronbach's alpha = 0.78 to 0.89), in patients with mood, anxiety or somatoform disorders (Cronbach's alpha = 0.81 to 0.91) and in patients with personality disorders (Cronbach's alpha = 0.76 to 0.91). In addition, the factor structure, as proposed in the manual, has a good fit and was shown to be invariant across various patient and community samples. The convergent and discriminant validity were considered satisfactory (de Beurs et al. 2009). In a similar sample of people with MID and BIF referred to specialised mental health care, the reliability, validity and factor structure of the DAPP‐SF were similar to those found in the previously mentioned samples (van der Heijden et al., submitted). In the current study, the reliability of the personality facets of the DAPP‐SF was acceptable to good for all three groups (MID: α = 0.66–0.90, BIF: α = 0.76–0.88, AVE: α = 0.63–0.93).

### 2.5 Statistical Analyses

All analyses were performed using IBM SPSS Statistics Version 29. Before conducting the main analyses, ANOVA (for continuous dependent variables) and chi‐squared tests (for nominal dependent variables) were conducted to determine the comparability of the groups regarding sex and age. Significant differences between the groups regarding these variables would be controlled for in the main analyses.

Concerning the main aim of the study, means and standard deviations of the personality trait facets were determined first. The assumptions of normality (skewness and kurtosis *Z*‐values < |1.96|were considered acceptable (Hair et al. 2022)) and homogeneity of variance (using Levene's test) were tested. Subsequently, multiple ANOVAs with simple contrast were performed to compare the personality trait facets of the MID group to those of the individuals from the BIF and AVE groups. The groups were entered as independent variables and the 18 personality trait facets of the DAPP‐SF as dependent variables. No correction for multiple testing was applied (*α* = 0.05), as correcting *α* would not reduce type‐I errors while testing for group differences on multiple dependent variables (García‐Pérez 2023). To determine effect sizes, partial eta‐squared (*η*_*p*_^2^) was used with the following criteria: 0.01 < *η*_*p*_^2^ < 0.06 small effect, 0.06 ≤ *η*_*p*_^2^ < 0.14 medium effect and *η*_*p*_^2^ ≥ 0.14 large effect (Richardson 2011).

## 3 Results

### 3.1 Preliminary Analyses and Descriptive Statistics of Personality Traits Facets

Preliminary analyses showed no differences in sex [*χ* ^2^(2) = 1.58, *p* = 0.453] and age in years, *F*(2,214) = 1.16, *p* = 0.317, between the MID group, BIF group and AVE group.

Table 2 displays means and standard deviations of the personality trait facets for each group. All facets showed a normal distribution of scores, except for the Callousness facet in the MID group and the Conduct Problems facet in the BIF group, which showed a high kurtosis. This highly peaked distribution in the Conduct Problems facet was also found in other samples (de Beurs et al. 2009) and was therefore accepted. The highly peaked distribution in the Callousness facet within the MID group was resolved by removing one outlier, after which the distribution of scores remained between the ranges for normality. Means and standard deviations displayed in Table 2 were adapted to the exclusion of this outlier. Preliminary analyses were redone to include the correct number of participants and remained nonsignificant. Levene's tests showed that homogeneity of variance could be assumed for all personality trait facets.

**TABLE 2:** Means and standard deviations of personality trait facets within each group.

Domains	MID group (*n* = 75)		BIF group (*n* = 69)		AVE group (*n* = 73)	
Trait facets	M	SD	M	SD	M	SD
Emotional dysregulation						
Affective lability	29.35	7.4	29.32	6.3	26.03	6.9
Anxiousness	21.57	5.5	22.51	5.0	19.11	4.8
Cognitive distortion	13.92	6.0	14.77	5.6	14.40	5.3
Identity problems	19.60	6.1	18.96	5.4	17.59	6.0
Insecure attachment	19.79	6.6	18.48	6.5	15.73	6.0
Narcissism	16.35	6.1	17.20	6.5	19.30	6.0
Oppositionality	28.05	8.8	29.19	7.1	28.73	8.8
Social avoidance	16.51	6.1	18.64	5.5	18.84	6.0
Submissiveness	23.51	8.1	25.13	6.9	24.66	7.3
Suspiciousness	20.29	7.0	21.00	7.3	17.64	7.9
Dissocial behaviour						
Callousness	17.85	5.4	19.48	6.3	18.62	5.9
Conduct problems	13.45	6.8	13.51	6.4	12.95	5.0
Rejection	17.64	5.6	19.45	6.3	18.62	6.3
Stimulus seeking	20.09	7.5	20.70	8.0	18.68	6.7
Inhibition						
Restricted expression	26.16	6.0	26.77	6.1	24.30	6.9
Intimacy problems	19.96	6.7	20.46	6.4	18.55	6.3
Compulsivity						
Compulsivity	24.88	8.1	23.58	6.9	23.86	6.1
Other						
Self‐harm	13.57	6.9	14.65	6.6	12.75	6.5

### 3.2 Comparing Personality Trait Facets of People With MID to the Other Groups

**FIGURE 1:**
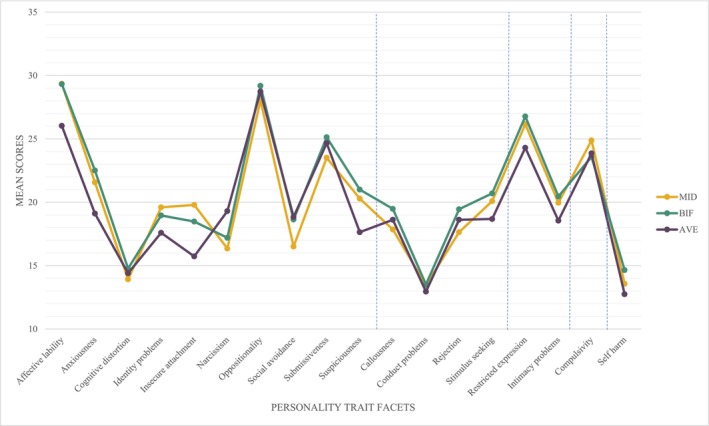
Personality profiles of people with MID, people with BIF and people with average educational levels (AVE).

Personality trait facets across the three groups are depicted in Figure 1, in which the dotted lines separate between the higher order personality trait domains. Visual inspection suggests that all three groups have similar line patterns. However, some larger differences between groups were seen in the personality trait facets Affective Lability, Anxiousness and Insecure Attachment, with participants with MID and participants with BIF scoring higher than the participants in the AVE group. The smallest differences were visible in the facets Cognitive Distortion, Oppositionality and Conduct Problems.

Using ANOVAs with simple contrasts, personality trait facets of the participants in the MID group were compared to those of the participants in the AVE group and the participants in the BIF group (see Table 3). Statistically significant contrasts were found in seven out of 18 personality trait facets when comparing the MID group and AVE group. Participants in the MID group showed significantly higher scores on Affective Lability, Anxiousness, Identity Problems, Insecure Attachment and Suspiciousness compared to the participants in the AVE group. The differences in Affective Lability, Anxiousness, Identity Problems and Suspiciousness showed a small effect and in Insecure Attachment a moderate effect. Participants in the MID group also scored significantly lower on Narcissism and Social Avoidance compared to the participants in the AVE group, both showing a small effect. There were no significant differences between the participants in the MID group and the participants in the BIF group, except for Social Avoidance, where the MID group scored lower than the BIF group, showing a small effect.

**TABLE 3:** Comparison of the MID group trait facets to those of BIF and AVE group.

Trait facets	ANOVA			Contrasts with AVE group^#^			Contrasts with BIF group^#^		
	*F*(2,214)	*p*	*η*_*p*_^*2*^	Difference	*p*	*η*_*p*_^*2*^	Difference	*p*	*η*_*p*_^*2*^
Emotional dysregulation									
Affective lability	5.58	0.004**	0.050	MID > AVE	0.004**	0.039			
Anxiousness	8.38	< 0.001***	0.073	MID > AVE	0.004**	0.038			
Cognitive distortion	0.41	0.666	0.004						
Identity problems	2.25	0.108	0.021	MID > AVE	0.039*	0.020			
Insecure attachment	7.70	< 0.001***	0.067	MID > AVE	< 0.001***	0.065			
Narcissism	4.45	0.013*	0.040	MID < AVE	0.004**	0.038			
Oppositionality	0.34	0.710	0.003						
Social avoidance	3.55	0.031*	0.032	MID < AVE	0.017*	0.026	MID < BIF	0.031*	0.022
Submissiveness	0.91	0.405	0.008						
Suspiciousness	4.11	0.018*	0.037	MID > AVE	0.031*	0.022			
Dissocial behaviour									
Callousness	1.38	0.254	0.013						
Conduct problems	0.19	0.829	0.002						
Rejection	1.62	0.200	0.015						
Stimulus seeking	1.39	0.253	0.013						
Inhibition									
Restricted expression	2.94	0.055	0.027						
Intimacy problems	1.68	0.190	0.015						
Compulsivity									
Compulsivity	0.68	0.509	0.006						
Other									
Self‐harm	1.41	0.245	0.013						

* *p ≤* 0.05,

** *p ≤* 0.01.

*** *p ≤* 0.001.

# Only significant contrasts are shown.

There were no significant differences between the participants in the MID group and the participants in the BIF group, except for Social Avoidance, where the MID group scored lower than the BIF group, showing a small effect.

## 4 Discussion

The aim of the current study was to compare maladaptive personality traits (i.e., facet scores) of people with MID referred to specialised mental health care to those of people with BIF referred to specialised mental health care and to those of people with average educational levels from a general mental health care setting. The results show small to no differences in personality trait facets between the sample of individuals with MID and those of the two comparison groups. Even though some personality trait facets showed mean level statistical differences between the groups, effect sizes were small. This means that the personality profiles in all groups point to similar types and severity of personality problems, which would lead to similar clinical decisions and treatment.

To date, little research has been done in which dimensional personality traits in people with MID were assessed, besides two previously mentioned studies (Haacke et al. 2025; Lie Sam Foek‐Rambelje et al. 2024) which are difficult to compare to the current results. In those studies, a clinician‐rated instrument (i.e., SWAP) was used comprising a different dimensional model of personality. Moreover, both studies combined samples of people with MID and BIF into one group. The study by Lie Sam Foek‐Rambelje et al. (2024) found differences in the Obsessionality domain between people with MID‐BIF and people with average educational levels. However, in the current study, no differences were found on the Compulsivity domain, which is quite similar to the Obsessionality domain. Regarding the domain Emotional Dysregulation, in the current study, we found some differences, albeit at a much smaller effect compared to the study by Lie Sam Foek‐Rambelje et al. (2024). The multiple small differences on the trait facets within the Emotional Dysregulation domain, as found in the current study, might have combined to a larger difference on the higher order domain of Emotional Dysregulation, as reported by Lie Sam Foek‐Rambelje et al. (2024). However, exploring the personality trait facets instead of personality domains offered a more nuanced representation of these differences, which is a notable strength of the current study.

### 4.1 Clinical Implications

The results of the current study show that individuals with MID referred to mental health care experience a high degree of (internalising) personality problems such as affective lability, oppositionality and restricted expression. As the extent of these personality problems is very similar to those of people with average educational levels referred to mental health care, these problems cannot simply be attributed to difficulties in intellectual or adaptive functioning or consequences thereof. For example, people with MID are often found to have some interpersonal difficulties, as described in the social domain of adaptive functioning (APA 2022). However, in the current study, the interpersonal dimensions, such as Restricted Expression, do not show to be different between groups. This might indicate that the DAPP, and by extension personality traits, actually measure different concepts from the adaptive problems seen in people with MID.

### 4.2 Strengths, Limitations and Future Research

The current study has some limitations that should be taken into account when interpreting the results. We used the DSM diagnoses of MID and BIF from the patient files to allocate individual participants to one of two groups. The additional information on which diagnoses were based was not included in the data, as a large variety of standardised assessments and additional observations and interviews were used to come to a DSM diagnosis of MID and BIF. Another limitation of the current study is that the differences in personality trait facets between groups were not controlled for by general psychopathology. As all participants were referred to primarily outpatient mental health services, it is assumed that all met the criteria for common mental disorders such as anxiety and depression, but also possible personality disorders. The type and severity of comorbid psychopathology might have differed between groups and therefore may have influenced the outcomes regarding differences in personality trait facets. Additionally, we are aware that the mode of administration of the instrument (interview‐administered) is prone to bias, and the simplification of the items in an average of 9% of items might have influenced the results. Even though these adaptations are in line with other research and clinical practice on the assessment of mental health problems in people with MID‐BIF (Kooijmans et al. 2022), the effects of these adaptations on results are not well researched (Jen‐Yi et al. 2015).

Future research should further explore the clinical utility of dimensional personality trait assessment in individuals with MID relative to those with higher levels of functioning, while controlling for general psychopathology and comorbid conditions. Given the minimal differences observed across groups, it is important to examine whether these findings can be replicated using alternative well‐validated dimensional personality instruments. Moreover, systematic evaluation of the implications of instrument adaptations (e.g., item simplification, interviewer administration) on personality assessment in people with intellectual disabilities is necessary.

### 4.3 Conclusions

The type and severity of personality trait facets of people with MID are quite similar to those of people with BIF or to those of people with average educational levels. This implies that clinicians should assess these personality trait facets in people with MID in a similar way to people with average educational levels. Even if some small textual adaptations or support are necessary for people with MID to complete these self‐report measures on personality trait facets, the results are equally informative as those of people with average educational levels. Consequently, this should lead to similar decisions and treatment of personality problems if the people with MID would not be excluded from mental health care due to high complexity on account of the co‐occurrence of intellectual, adaptive and behavioural problems (Pinals et al. 2022). The results of this study imply that clinicians should look beyond the intellectual and adaptive disabilities when assessing for mental health problems in people with MID, while meeting their needs when it comes to the treatment of these problems. Clinicians and researchers are to improve their knowledge and skills regarding the assessment and treatment of maladaptive personality traits in people with MID, to reduce exclusion from research, assessment and treatment.

## Conflicts of Interest

The authors declare no conflicts of interest.

## Data Availability

The data that support the findings of this study are available from GGZ Oost Brabant. Restrictions apply to the availability of these data, which were used under license for this study. Data are available from the author(s) with the permission of GGZ Oost Brabant.
